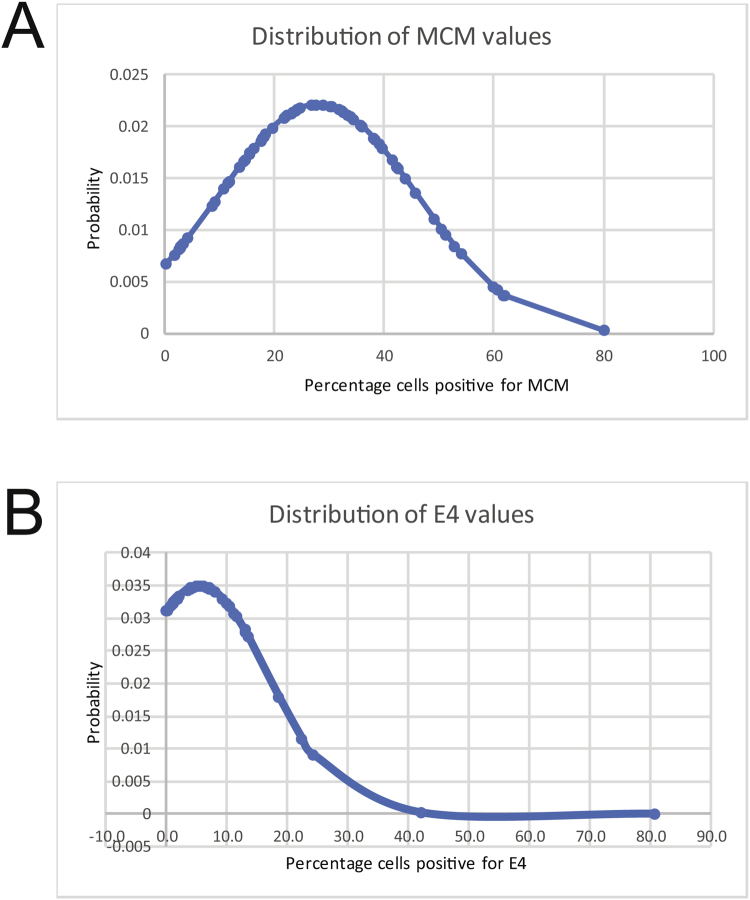# Risk stratification of cervical disease using detection of human papillomavirus (HPV) E4 protein and cellular MCM protein in clinical liquid based cytology samples

**DOI:** 10.1016/j.jcv.2018.08.011

**Published:** 2018-11

**Authors:** Andrew Stevenson, Kim Kavanagh, Jiafeng Pan, Lynne Stevenson, Heather Griffin, John Doorbar, Evelyn Scott, Miriam Deeny, Kate Cuschieri, Sheila V. Graham

**Affiliations:** a MRC-University of Glasgow Centre for Virus Research, Institute of Infection, Immunity and Inflammation, College of Medical, Veterinary and Life Sciences, University of Glasgow, Garscube Estate, Glasgow, G61 1QH, Scotland, UK; b Scottish HPV Reference Laboratory, Royal Infirmary of Edinburgh, 51 Little France Crescent, Edinburgh, EH16 4SA, Scotland, UK; c Department of Pathology, University of Cambridge, Tennis Court Road, Cambridge, CB2 1QP, UK; d Mathematics and Statistics, Livingstone Tower, University of Strathclyde, Glasgow G1 1XQ, Scotland, UK; e Stobhill Hospital, 133 Balornock Rd, Glasgow G21 3UW, Scotland, UK; f Veterinary Diagnostic Services, School of Veterinary Medicine, College of Medical, Veterinary and Life Sciences, University of Glasgow, Garscube Estate, Glasgow, G61 1QH, Scotland, UK

**Keywords:** HPV, human papillomavirus, CIN, cervical intraepithelial neoplasia, IHC, immunohistochemistry, LBC, liquid based cytology, MCM, minichromosome maintenance, HPV, Cervical intraepithelial neoplasia, Biomarkers, Liquid based cytology, Cytospin

## Abstract

**Background:**

While human papillomavirus (HPV) DNA testing offers high sensitivity for the detection of significant cervical disease, its specificity is suboptimal given the high prevalence of transient HPV infections (CIN1 or less). Biomarkers to identify those suffering from low grade disease from those with high grade disease could save healthcare costs and reduce patient anxiety.

**Objective:**

The objective of the present work was to develop and test an immunohistochemistry (IHC)-based dual viral and cellular biomarker strategy which was applicable to liquid based cytology (LBC) samples.

**Study design:**

We developed a novel IHC assay for detection of HPV E4 and cellular minichromosome maintenance (MCM) proteins in routinely taken cervical LBC samples using cytospin-prepared slides. The assay was applied to a prospective cohort of Scottish women referred to a colposcopy clinic due to preceding cytological abnormalities. The performance of the biomarkers for detection of clinically insignificant (CIN1 or less) versus significant disease was determined.

**Results:**

A total of 81 women were recruited representing 64 cases of <=CIN1 and 28 of CIN2 + . Biomarker performance relative to histopathology outcomes showed high levels of MCM detection was significantly associated with CIN2+ (p = 0.03) while E4 was detected more frequently in <=CIN1 (p = 0.06).

**Conclusions:**

Combined detection of a host proliferation marker and a marker of viral gene expression could allow triage of cases of clinically insignificant disease prior to colposcopy. However, there was overlap between distributions of MCM levels in CIN2+ and <=CIN1 suggesting that additional biomarkers would be required for improved specificity. Combined with cytospin-prepared slides this approach could provide a means of risk stratification of disease in low resource settings.

## Highlights

• Cytospinning is a viable method for preparing LBC cells for antibody staining.
• We assessed the performance of a dual biomarker (one viral: HPVE4, one cellular: MCM2) in risk stratification of cervical disease.
• MCM2 was significantly associated with CIN2+ (p = 0.03). HPVE4 was associated with CIN1/normal (p = 0.06).
• The dual biomarker approach may be useful to risk stratify cervical disease especially in resource-poor settings.

## 1 Background

Clinically insignificant cervical disease represents transient, asymptomatic HPV infections that are cleared by the immune system [1]. Clinically significant infections are not cleared/not detected by the immune system leading to persistent infections that can progress to cancer [2,3]. The high prevalence of clinically insignificant HPV infections, particularly in young women, means the HPV DNA tests in clinical use can lack specificity because they detect presence or absence of viral nucleic acid but cannot measure virus activity. Virus activity is the expression of the virus genome and the impact this has on host genome expression, and has potential to be a sensitive and specific indication of cervical disease [4,5]. New diagnostic approaches to identify transient versus significant HPV infection would improve the management of associated diseases immensely.

The infectious HPV life cycle is tightly linked to the differentiation process of the host epithelium [3]. HPV infects actively dividing basal epithelial cells and establishes its episomal genome at low copy number in the nuclei of these cells. Infected basal cells can move out of the basal layer as transit amplifying cells, which undergo keratinocyte terminal differentiation. During this process, the virus displays a highly orchestrated gene expression programme that responds to keratinocyte differentiation. Viral E6 and E7 proteins stimulate progression of the cell cycle from G1 into S-phase in order to allow replication of viral genomes [5]. In the mid layers of the epithelium, increased synthesis of HPV E1 and E2 proteins allows viral genome replication and HPV E4 protein, the most abundant viral protein, is synthesised and facilitates viral replication and virion egress.

Most HPV infections are cleared by the immune system. However, if an infection becomes persistent in the epithelium, the host and viral genomes undergo alterations that make progression to preneoplastic and neoplastic disease more likely. Key among these changes due to persistence is increased expression of HPV E6 and E7 proteins leading to increased levels of cell cycle proteins such as p16, Ki67 and MCM. [3,6,7]. Therefore, increased viral E6 and E7 expression is a feature of persistent, clinically significant disease. However, these proteins are hard to detect in < CIN3 due to low expression levels in non-persistent (i.e. normal) HPV infections. Instead, surrogate biomarkers of E6/E7 activity are used. p16 is considered the best validated “gold standard” for detecting increased HPV E6 and E7 activity in clinical samples [8,9] but MCM, a cellular marker of DNA replication, is an alternative surrogate biomarker [[10], [11], [12]]. Previous studies revealed that increased MCM (as a surrogate for E7) was a potential biomarker of clinically significant disease [11,12]. In contrast, E4 is a potential biomarker of clinically insignificant disease because in an analysis of formalin-fixed paraffin-embedded tissues, its expression levels were greatest in CIN1 and lowest in CIN3 [12], and there are excellent antibodies against E4 [13].

## 2 Objectives

Biomarker-based risk-stratification of HPV infection is a key priority for development given the increasing move to cervical screening programmes based on primary HPV testing. A single biomarker may be less optimal for disease stratification compared to a biomarker combination/matrix. A matrix which incorporates both a viral and a cellular target may confer sensitivity and specificity respectively. Here, we describe an initial investigation into the performance of a dual biomarker based on IHC-based detection of E4 and MCM in routinely taken liquid based cytology (LBC) to determine/stratify underlying disease.

## 3 Study Design

### 3.1 Clinical samples

LBC samples were collected with informed consent from a cohort of 81 women referred to a colposcopy clinic in the west of Scotland. LBC samples were allocated a study number in the clinic unrelated to any patient identifiers, stored at 4 °C and transferred to the research laboratory.

### 3.2 Disease ascertainment

Disease was defined by histopathology results: with significant/high grade defined as histology-confirmed CIN2+, insignificant/no disease defined as histology confirmed <=CIN1 or no clinically indicated biopsy due to no abnormality detected on colposcopy.

### 3.3 HPV genotyping

HPV genotyping was with the Optiplex HPV Genotyping Assay (DiaMex, Heidelberg, Germany) according to manufacturer’s instructions. This PCR based test with luminex technology can resolve 24 HPV types (HPV6, 11, 16, 18, 26, 31, 33, 35, 39, 42, 43, 44, 45, 51, 52, 53, 56, 58, 59, 66, 68, 70, 73, 82) including all HR-HPV types as defined by the International Agency on Cancer.

### 3.4 Cell culture

HeLa cells were grown in Dulbecco’s modified eagles medium (Invitrogen), 10% foetal calf serum (Invitrogen) and penicillin (50 U/ml)/ streptomycin (50 μg/ml) (Invitrogen), at 37 °C in a 5% CO_2_ humidified incubator. Transfection with E4 expression plasmid pMV11 was with Lipofectamine 2000 according to the manufacturer’s instructions.

### 3.5 Cytospin and ThinPrep

LBC samples (100 μl) in PreservCyt Solution (Hologic) were deposited on Klinipath plus slides using a Shandon 3 Cytospin (450 rpm, 10 min). Slides were fixed for 10 min in 10% buffered formalin and washed twice in buffer TBS-T (10 mM Tris−HCl, pH7.5, 10 mM EDTA, 100 mM NaCl + 1% (v/v) Tween-20). Thinprep slides were prepared using the ThinPrep T2000 (Hologic) using 20 ml LBC samples in PreservCyt Solution (Hologic). Slides were briefly placed in 95% (v/v) ethanol and treated with Cytofix (Cellpath), immersed in 50% (v/v) ethanol for 10 min and fixed for 10 min in 10% buffered formalin, followed by washing in TBS-T.

### 3.6 Immunohistochemistry

Heat-induced epitope retrieval was carried out in 10 mM sodium citrate buffer, pH 6.0 using a Menarini Access Retrieval Unit, at 110⁰C on full pressure for 10 min. Slides were loaded on to a Dako Autostainer, rinsed in TBS-T, blocked in Dako Real TM peroxidise-blocking solution for 5 min followed by washing in TBS-T. Slides were incubated for 30 min in primary antibodies diluted in Dako universal diluent (1/800 for monoclonal antibody FH1.1 reactive against E4 from HPV types 16, 18, 31, 33, 35, 39, 45, 51, 52, 53, 56, 58, 59, 66, 67, 70 (a gift from John Doorbar [13,14])) and 1/100 for MCM2 rabbit polyclonal antibody (Abcam ab31159). Following two washes in TBS-T, slides were incubated with secondary antibody for 30 min (Dako K4001and K4003), washed twice in TBS-T, incubated for 10 min in Dako K5007 DAB followed by three washes in water. Slides were counterstained with Gills Haematoxylin, dehydrated, cleared and then mounted in synthetic resin.

### 3.7 Optimisation of staining and acceptance criteria

At least 3 photographs of stained cells were taken across each slide. Images were input into Photoshop. One individual determined total cell numbers in each field of view by counting haematoxylin-stained nuclei, then MCM or E4 positive cells were counted by two different individuals independently blind to the clinical data. Averages of the two counts were taken for further analysis. Most samples contained intact cells and some cell fragments. Cytobrush acquisition of samples can damage cells. We counted staining of only intact cells because E4 is a cytoplasmic stain while MCM is a nuclear stain and we wished to be able to compare levels of E4 and MCM-stained cells in the sample cohort. Some LBC samples contained clumps of material that non-specifically trapped antibody. Therefore, samples were filtered using a cell strainer size 70 μm (Corning CLS431751 SIGM).

### 3.8 CINtec

Thinprep slides were stained using the Roche CINtec®Plus system on the Roche Ultra immunohistochemistry platform (Roche MTM Laboratories) from residual LBC material. CINtec PLUS interpretation was blind to other results. Samples with one or more cervical epithelial cells that simultaneously showed brown cytoplasmic immunostaining (p16) and red nuclear immunostaining (Ki-67) were classified as positive regardless of the morphologic appearance of the cells. Slides with excessive background staining were considered not evaluable and excluded from analysis.

### 3.9 Evaluation of clinical performance of MCM and E4

Basic descriptive statistics stratified by grade of disease were produced and the distribution of both MCM and E4 values were visualised in boxplots. Differences in E4 and MCM levels by disease grade were evaluated using logistic regression were the outcome variable was high-grade vs low-grade/normal disease. Finally MCM and E4 were considered together by examining the correlation between the measures and examining if this differed by disease group. Both MCM and E4 were considered together in a multivariable logistic regression model. This approach was used to access the association between biomarker and disease grade because the numbers in each disease grade were quite low for splitting into training and test data sets for building and testing a prediction model.

## 4 Results

The aim of the study was to determine the performance of E4, a biomarker of transient HPV infection (≤CIN1), in combination with MCM2, a biomarker of significant cervical disease in a cohort of patients who had an abnormal Pap smear test result, were called to attend a colposcopy clinic, and tested positive for HPV.

### 4.1 Confirmation of analytical performance of MCM and E4 antibodies

**Fig. 1:**
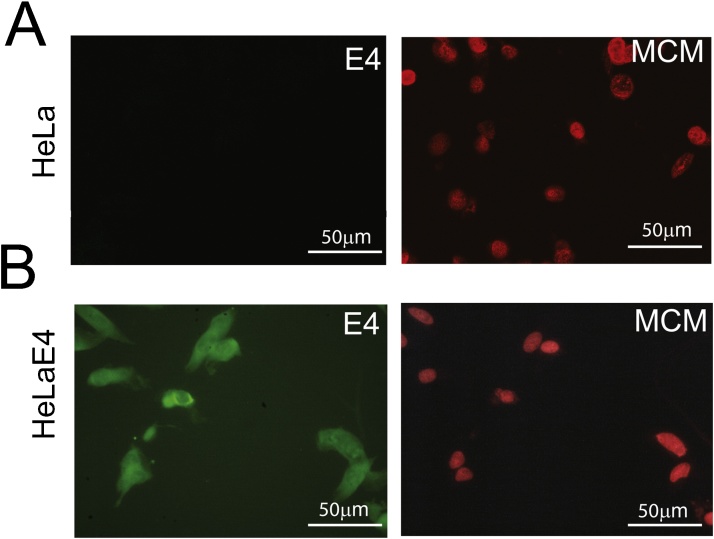
**Validation of E4 and MCM antibody staining in cytospin cell preparations**. A. HeLa cells or B. HeLa cells transfected with an expression plasmid for HPV16 E4 were grown and harvested in PreservCyt. cell populations were deposited on slides by cytospin, stained with antibodies against MCM or E4 and visualised using immunofluorescence microscopy.

To test specificity of the antibodies for cellular MCM and viral E4 proteins, HeLa cells, and HeLa cells transfected with an expression plasmid for HPV16 E4 were suspended in PreservCyt, stained with E4 and MCM antibodies and visualised by immunofluorescence microscopy. Fig. 1A shows E4 detection throughout the HeLaE4 cells, but not the untransfected HeLa cells, while nuclear MCM was detected in both cell types as expected (Fig. 1B).

### 4.2 Testing E4 and MCM detection by IHC on ThinPrep versus cytospin slides

**Fig. 2:**
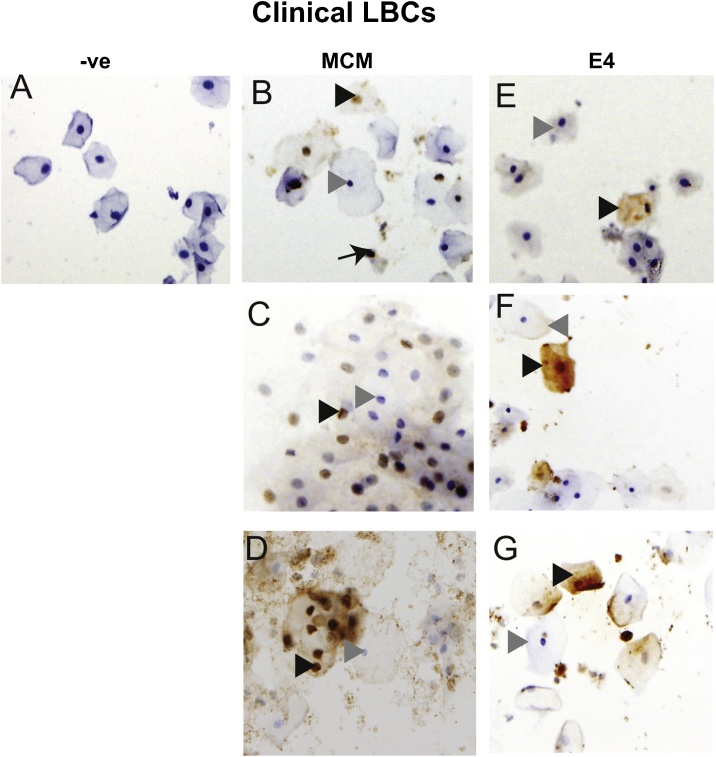
**Criteria for staining positivity on LBC cells. A.** –ve, negative control staining. B-DMCM2 staining showing brown-stained nuclei (black arrowheads). Unstained nuclei are blue due to the hematoxylin counterstain (gray arrowheads). B. a typical field of view from the sample cohort showing several MCM-stained nuclei. C. Many packed cells showing variability in strength of MCM staining. D. Some samples contained debris which trapped antibody. However, clear positive brown-stained nuclei and negative blue-stained nuclei were visible. The arrow in B. indicates a nucleus with partial cytoplasm. To ensure that only intact cells were counted, such cells were excluded from the counts. E–G, E4 staining showing brown-stained cytoplasm (black arrowheads). Unstained cells are shown with blue nuclei due to the hematoxylin counterstain (gray arrowheads). E. a typical field of view from the sample cohort showing one E4 stained cell. F. E4 staining was stronger in some samples. G. E4 staining was sometimes seen in only a portion of the cytoplasm and is likely due to how cells were deposited on the cytospin slides.

We compared cytospin centrifugation to the ThinPrep method (T2000) to determine the best slide preparation for IHC analysis using an initial cohort of 20 LBC samples. The same antigen retrieval protocol was used for each slide type. Cytospin performed better than Thinprep because adherence of LBC cells on the ThinPrep-prepared slides was poor following the antigen retrieval protocol required for efficient immunostaining with the selected biomarkers. For cytospin-prepared slides, only 100 μl of each LBC sample was required thus ensuring that multiple biomarkers could be tested on a single sample. Finally, cytospin deposits two cell populations on a single slide meaning that an antibody-negative control could be included on each slide (Fig. 2A). Cells were counted positive for E4 if the marker was detected in the cytoplasm (Fig. 2E-G), and for MCM if staining was in the nucleus (Fig. 2 B–D). Three images are shown for each biomarker stain to represent the range of staining obtained.

### 4.3 Description of clinical cohort and underlying pathology

81 patients referred to a colposcopy clinic in the West of Scotland as a consequence of preceding cytological abnormalities were consented to the study and an LBC sample was taken in the clinic from each patient for the study. The average age of the cohort was 31 years (range 20–60 years). HPV was detected in 78/81 (96.3%) samples with HR-HPV detected in 66/81 (81.5%) samples (Supplementary Table 1). One third of all study participants were infected with HR-HPV16 and/or 18 (27/81, 33.3%). Following histopathological examination, there were 18 cases of CIN2+, 40 cases of CIN1 and 22 were designated “normal” or “nil” or “no biopsy”. All CIN2+, 38/40 CIN1 and all “normal” cases were positive for HR-HPV. Only HR-HPV positive samples were included in the analysis.

### 4.4 Overall MCM and E4 positivity in LBC samples

Of the 81 clinical samples, 73 and 71 respectively had sufficient intact cells to allow MCM and E4 antibody staining. All 73 samples examined were positive for MCM (90.1%). Of the 71 samples that had sufficient cell numbers, 45 (63.4%) were positive for E4. The percentage of cells within a sample positive for MCM ranged from 3 to 86.7% with an average of 27.5%. MCM will normally be expressed in any cell undergoing cell cycle, e.g. in epithelial stem cells or in cells during wound closure. Therefore, there may always be some MCM expression in cervical tissue so we decided not to count as positive any sample with any MCM detection. An arbitrary limit of >20% of cells stained within a cell population was chosen as “high” (or positive) MCM because this was just below the average value and on the upward shoulder of a normal distribution plot of MCM values (Supplementary Fig. 1). The range of cells in any sample positive for E4 was 0 to 80.6% with an average of 5.5%. E4 staining appeared to fall into two classes, no, or very few cells stained (43 samples), or >5% (28 samples) of cells stained. For these two reasons samples were designated “high” E4 if staining was detected in ≥ 5% of cells.

### 4.5 Does biomarker matrix detection correlate with histological disease status?

**Fig. 3:**
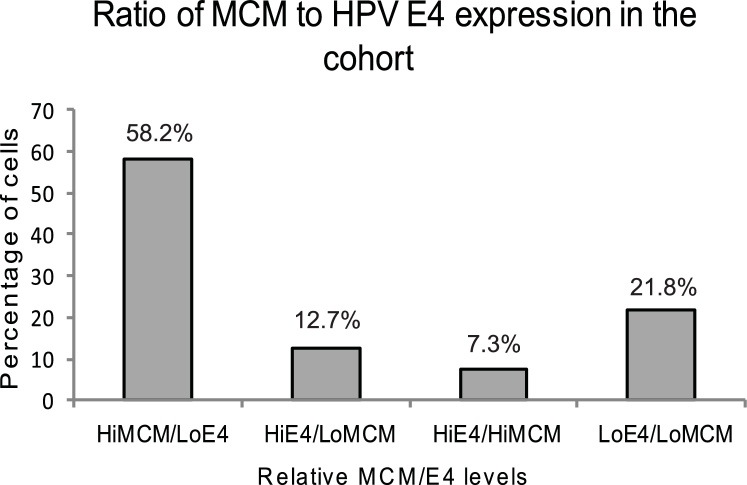
**Categories of ratios of MCM and E4 staining.** Graph of relative MCM and E4 levels versus percentage of cells in each cell population evaluated. HiMCM, >20% MCM positivity. LoMCM, <20%MCM positivity. HiE4, ≥5% positivity. LoE4 < 5% positivity.

**Fig. 4:**
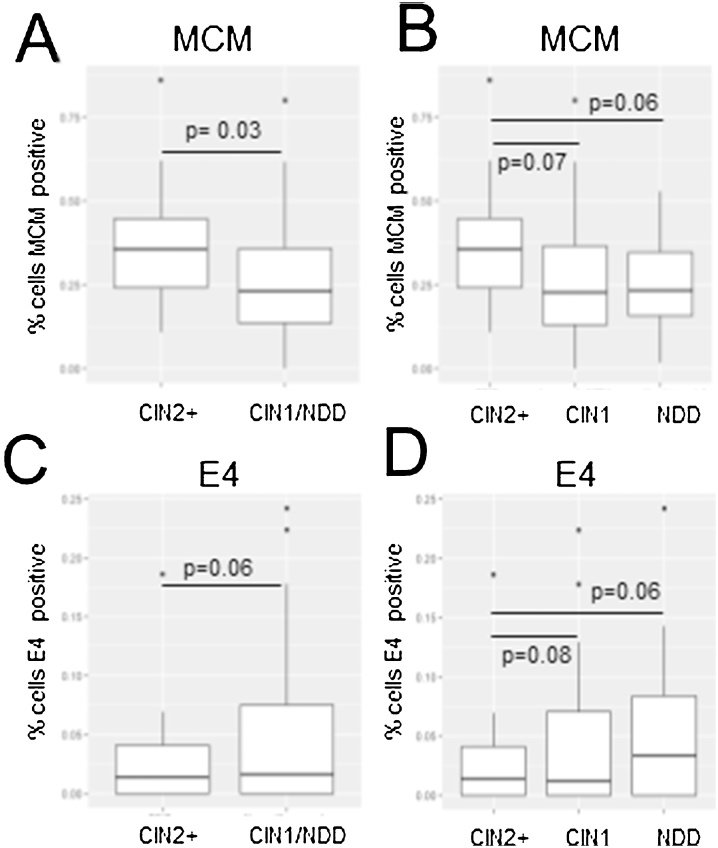
**Percentage LBC cells positive for the biomarkers MCM and E4 versus histopathological grade of disease**. A. MCM2 positivity considering CIN2+ and <=CIN1. B. MCM2 positivity considering CIN2+, CIN1/HPV + ve and normal/nil/no biopsy taken. C. E4 positivity considering CIN2+ and <=CIN1. D. E4 positivity considering CIN2+, CIN1/HPV + ve and normal/nil/no biopsy taken. The dots above the boxes on the plots are outlier values. p-values are shown above the boxes on the plots.

69 samples analysed were positive for HR-HPV that could be detected with the pan-E4 antibody. There was a segregation of E4 and MCM staining in our clinical cohort: 70.9% of samples had either high MCM/lowE4 (58.2%) or high E4/lowMCM (12.7%) staining. Of the remainder, 7.3% had high E4/high MCM and 21.8 had low E4/low MCM (Fig. 3). MCM positivity (>20% of cells stained) was statistically more likely to be present in CIN2+ (p = 0.03). Conversely, E4 detection (≥ 5%) was more frequent in CIN1 and normal/nil/no biopsy but just failed to reach statistical significance (p = 0.06). MCM marked LBCs graded CIN2+ when compared with low grade/normal combined (Fig. 4A), or low grade or normal separately (Fig. 4B). E4 levels were very similar in combined low grade/normal samples compared with CIN2+ (Fig. 4C) but were greater in samples graded as histologically normal (but HPV-positive) compared to low grade or CIN2+ (Fig. 4D). p-values were calculated for MCM and E4 detection considering CIN2+ versus CIN1 and CIN2+ versus normal and are shown on the graphs (Fig. 4C, D). None were less than or equal to p = 0.05 due to the small numbers in each group.

### 4.6 Comparison of MCM detection in cytospin-prepared slides with p16 detection using CINtec

The ability of MCM detection in cytospin-prepared slides to identify significant disease was compared to the detection of p16/Ki67 using CINtec. Of the 73 MCM-positive samples, 12 were excluded from analysis due to either insufficient cells or unsatisfactory results. Sensitivity was 1.00 while specificity was 0.404. Data from the remaining 61 LBCs showed an accuracy of 0.639 comparing cytospin MCM detection with CINTec positivity. MCM detection showed a sensitivity of 0.651 and a specificity of 0.611 at detecting high grade disease.

## 5 Discussion

Since its introduction, cervical screening via cytology has reduced incidence of cervical cancer [15]. However, cytology is inherently subjective with variable performance across settings. Molecular HPV testing is becoming the optimal method of primary cervical screening [16] and has been used, successfully as a “test of cure” of treatment [17]. While the sensitivity, negative predictive value and objectivity of molecular HPV tests are good, they cannot indicate if a transient infection can progress towards clinically significant disease. Consequently, biomarkers to risk-stratify HPV infection are required. In particular, a biomarker strategy that could identify those lesions that are unlikely to progress to cancer would reduce healthcare costs and clinic attendance time and anxiety for patients.

p16/Ki67 are robust biomarkers of high grade disease and have been assessed as a triage of primary HPV positivity in primary screening [[18], [19], [20]]. Like p16 and Ki67, cellular MCM levels respond to increased expression of viral oncoproteins E6 and E7 that mark clinically significant disease [12]. This is due to the central role of MCM2 in DNA replication licensing [21], a process that is activated aberrantly by HPV E6/E7 expression [22,23] [24]. A commercial assay is currently available to detect this protein, together with another DNA replication marker TOP2A, in blocks of formalin-fixed cervical lavage cells [11]. Conversely, HPV E4 marks clinically insignificant disease [12,13]. This is because HPV E4 protein is expressed during the late stage of productive HPV infections [10], which are not present, or present only at a very low level, in CIN2+ [12]. In CIN2+, (except for E6 and E7 proteins, whose expression is increased) expression of all viral proteins is disrupted [2,3,6]. Therefore, E4 should be detected in HPV-positive CIN1 or less. This hypothesis has been proved by analysis of tissue sections representing different grades of cervical disease [12]. Previous studies indicated that these complementary biomarkers of cervical disease could have prognostic capabilities [12]. Staining of formalin-fixed paraffin-embedded CIN1 lesions showed that the majority of cells in the tissues stained positive for either E4 or MCM and only a few cells showed dual staining. In CIN2+ high levels of MCM staining were detected but E4 was rarely detected. Analysis of tissue sections can reveal the pattern of biomarker expression and allow some quantification of levels of expression. However, it is difficult to adapt for high throughput settings such as screening.

LBCs are a suitable biospecimen for high-throughput contexts. However, cell numbers in individual samples can vary widely and the sample can contain HPV-infected and uninfected cells. Moreover, the cells in the sample can represent a range of disease stages if these are present across an individual cervix. Consequently, percentage positivity for either biomarker assessed in the present study likely gives only an estimate of their levels in-situ. Our choice of cut-off values for MCM and E4 could be inappropriate given the small number of samples tested. It will be useful in a future study with greater numbers of samples to compare data with and without cut-off values. Despite these caveats, as predicted by the previous tissue-based study, high MCM detection was associated with CIN2+ and E4 association with <=CIN1 was close to significance. E4 expression levels can be very variable in CIN1 lesions [12] and, as a cytoplasmic protein it may be more likely than a nuclear protein to leach out of LBC cells during storage and processing. However, the low numbers of samples in the study will impact the significance of the E4 results. Some E4 was detected in histopathologically-defined “normal” samples. These were HR-HPV positive and therefore likely represent transient infections. A further study using a larger cohort is required to validate E4 as a biomarker of clinically insignificant disease in LBC samples, or conversely, that low or no E4 expression is a biomarker of CIN2 + . A previous study found that detection of p16 but no detection of viral L1 protein was sufficient to predict CIN3 [25]. It would be useful to investigate a quadruple biomarker matrix incorporating p16, MCM, E4 and L1 in an LBC-based cervical disease risk-stratification test. However, biomarker analysis is not straightforward. For example, although p16 and MCM are biomarkers of clinically significant disease, the former is found in CIN1 and CIN2+, albeit with a different distribution, and is present in senescent cells while the latter can be present during metaplasia or inflammation. Detection of the viral biomarkers E4 and L1 seems to more restricted to ≤ CIN1 [12,13]. Thus combinations of markers, especially when particular patterns are pathognomonic of disease, should offer clear benefits.

In the developed world, a matrix test could be used as an adjunct to HPV DNA testing to determine HPV activity in a lesion and predict the probability of disease progression in primary screening, low-grade triage or test of cure contexts. Approximately 70% of women who are HR-HPV positive at primary screen are cytology negative and this group represents a management challenge. A biomarker approach (either stand-alone or adjunctive to cytology) which could mitigate against the subjectivity of cytological observation and allow separation of low-grade disease from significant CIN would prove useful. In future, chromogenic *in situ* HPV DNA staining could indicate how many LBC cells in a sample are infected so MCM or E4 positivity could be calculated on HPV-positive cells only. Excellent antibodies against MCM are commercially available. The pan-E4 antibody is available from DDL Diagnostics Laboratory, The Netherlands [14]. It would also be important to know whether LBC samples from different sources stain with the selected antibodies to determine if transport or storage affect the protocol. Such technical optimisation, within the context of a wider prospective study where MCM and E4 are considered as stand-alone markers and as an adjunct to cytology, will be of value. Further assessment of the technical and clinical performance of the E4/MCM test would be required in longitudinal field studies. It will be important in future to compare this test with alternative strategies such as OncoE6 [26], which risk stratifies high grade disease, or cytoactivL1 that risk stratifies low grade disease [27].

## 6 Conclusion

In this “proof of principle” study we have shown that MCM and E4 are a promising biomarker matrix for the separation of disease grade in routinely taken cervical samples. MCM can identify CIN2+ when used in the cytospin technique. Although E4 was detected in LBC cells, its usefulness as a biomarker of clinically insignificant disease requires further investigation in a larger LBC cohort. The cytospin approach could prove useful in low and middle income settings lacking infrastructure for standardised cytology and high-throughput HPV testing.

## Author Contributions

SVG designed and directed the study and wrote the paper. AS, KC, ES and MD acquired the data. SVG, AS, KK and JP analysed the data. JD and HG helped conceive the approach. KC, JD and MD helped draft the article. All authors approved the final submitted version of the manuscript.

## Funding

This work was a sub-project awarded to SVG funded by a Medical Research Council Confidence in Concept MC_PC_13063 award.

## Ethical approval

REC reference 12/SS/0108 was granted by the Southeast Scotland Research Ethics Committee.

## Conflict of interest

SVG, AS, MD, ES, JP, KK, LS, JD and HG declare no conflict of interest. KC’s institution has received grant monies and/or consumables from the following companies in the last three years: Hologic, Cepheid, Qiagen, Euroimmun, LifeRiver, Genefirst and SelfScreen.

## Figures and Tables

**Figure:**