# Supplementary material for: Risk stratification of cervical disease using detection of human papillomavirus (HPV) E4 protein and cellular MCM protein in clinical liquid based cytology samples
Source: J Clin Virol. 2018 Nov;108:19–25. doi: 10.1016/j.jcv.2018.08.011 (PMC6224362; doi:10.1016/j.jcv.2018.08.011)
Supplement: Supplementary file 1 [file mmc1.docx]

**Supplementary Figure 1.** Normal distribution curves of percentage cells in individual samples positive for the biomarkers A. MCM2 or B. HPVE4.
