# Supplementary material for: Risk stratification of cervical disease using detection of human papillomavirus (HPV) E4 protein and cellular MCM protein in clinical liquid based cytology samples
Source: J Clin Virol. 2018 Nov;108:19–25. doi: 10.1016/j.jcv.2018.08.011 (PMC6224362; doi:10.1016/j.jcv.2018.08.011)
Supplement: Supplementary file 2 [file mmc2.docx]

**Supplementary Table 1. HPV genotyping of the clinical samples and associated disease.** HR-HPV detection (13 genotypes designed by IARC as group 1 carcinogens) is indicated in grey cells. ND= not detected. HPV genotypes in bold type are those detected by the pan E4 FH1.1 antibody used in this study. NDD= histological diagnosis of normal/no biopsy/nil. The samples where HPV genotype was not available was excluded from the study.

| **Sample ID** | **HPV genotype** | **Disease**  **status** | **Sample ID** | **HPV genotype** | **Disease**  **status** |
| --- | --- | --- | --- | --- | --- |
| CIC001 | 26, **33** | CIN2+ | CIC042 | 6, **45, 51, 53**, 70 | CIN1 |
| CIC002 | **16, 18, 66** | CIN1 | CIC043 | **31** | CIN1 |
| CIC003 | **16** | NDD | CIC044 | **39** | CIN1 |
| CIC004 | **52, 53, 56** | CIN1 | CIC045 | **16**, 44 | CIN2+ |
| CIC005 | **39** | NDD | CIC046 | **45,66** | NDD |
| CIC006 | **51** | NDD | CIC047 | **16**, 44 | CIN1 |
| CIC007 | 6, 42, **52, 56** | NDD | CIC048 | **45, 66** | CIN1 |
| CIC008 | **39** | NDD | CIC049 | 43, **45, 58, 59** | CIN1 |
| CIC009 | 26, **33, 70**, 82 | CIN1 | CIC050 | **52, 66** | CIN1 |
| CIC010 | 11,**16** | NDD | CIC051 | **16** | NDD |
| CIC011 | **51, 56** | NDD | CIC052 | 26, **66** | NDD |
| CIC012 | **16** | NDD | CIC053 | 73 | Not available |
| CIC013 | **51, 53** | CIN1 | CIC054 | ND | NDD |
| CIC014 | **16**, 42 | NDD | CIC055 | **51, 52** | NDD |
| CIC015 | **53** | NDD | CIC056 | **51** | CIN1 |
| CIC016 | ND | CIN1 | CIC057 | **16, 31** | CIN2+ |
| CIC017 | **16** | NDD | CIC058 | 42, **59** | CIN1 |
| CIC018 | **45, 52, 56, 66** | CIN1 | CIC059 | **16**, 42, **52, 58** | CIN2+ |
| CIC019 | 59**, 66** | NDD | CIC060 | **16, 31, 39** | CIN1 |
| CIC020 | 44, **56**, 59, **66** | NDD | CIC061 | **33**, 42 | CIN2+ |
| CIC021 | **16, 58** | CIN2+ | CIC062 | **39** | CIN1 |
| CIC022 | **33**, 82 | CIN1 | CIC063 | **31** | CIN2+ |
| CIC023 | **16, 31** | CIN1 | CIC064 | **18** | CIN1 |
| CIC024 | 59, **66** | NDD | CIC065 | **16, 31** | CIN2+ |
| CIC025 | 42, **73** | CIN1 | CIC066 | ND | NDD |
| CIC026 | **39**, 42 | CIN1 | CIC067 | **16, 18** | CIN1 |
| CIC027 | 42, **59**, 82 | NDD | CIC068 | **39** | CIN1 |
| CIC028 | 6, **66, 70** | CIN1 | CIC069 | **66** | CIN1 |
| CIC029 | **16,** 82 | CIN1 | CIC070 | **16** | CIN1 |
| CIC030 | **53** | CIN1 | CIC071 | **51, 53** | CIN1 |
| CIC031 | 6, **16, 31** | CIN2+ | CIC072 | **52** | CIN1 |
| CIC032 | **16, 18, 31, 66** | CIN2+ | CIC073 | **16, 35** | CIN2+ |
| CIC033 | **56** | CIN2+ | CIC074 | **16** | CIN1 |
| CIC034 | **16, 45, 53, 59** | CIN2+ | CIC075 | **18, 53** | CIN1 |
| CIC035 | **16** | CIN2+ | CIC076 | invalid sample | CIN1 |
| CIC036 | **51, 53** | CIN1 | CIC077 | 73 | CIN1 |
| CIC037 | **33** | CIN2+ | CIC078 | **18** | CIN1 |
| CIC038 | 82 | CIN2+ | CIC079 | **53** | CIN1 |
| CIC039 | 44, **45, 56** | NDD | CIC080 | **39** | **NDD** |
| CIC040 | **52** | CIN2+ | CIC081 | 42 | CIN1 |
| CIC041 | **58**, 82 | CIN1 |  |  |  |
